# Indents

**Published:** 1907-06-15

**Authors:** 


### INDENTS.

☞ **Brief Articles of Technical or Scientific Value will receive notice and comment. Contributions invited.**

**Lower Impressions.** Warm compound to a consistency at which it will adapt itself readily to the shape of the mouth. Place it upon the tray, put in the mouth and pressed down *almost* as far as you desire it to go; then wait for a space of a minute or two until it hardens slightly.

During this interval of time I have my assistant direct a current of cold air into the mouth from a rotary fan in the dental engine; this chills the outer layer of the compound, and prevents it from flowing away from the jaw when pressure is put upon it. While the layer of impression material that is in contact with the tissues is still warm and plastic, press the tray down just a little further, and then hold it steadily with about half the force used in pressing it down the last time, until it is quite hard; then remove the tray and pour the impression.

In order that we may know why we proceed in this manner, let us go over the taking of this impression again. In the first place, when the material is put in the mouth and pressed down, we get an ordinary impression; after it is allowed to stand a little time, the outside chilled and pressed down a second time, then the impression is changed from the ordinary impression, but in what way?

When pressing down on the tray the second time the hard ridge of the alveolus cuts just a little deeper into the impression material and remains practically unchanged, while the soft tissues down on the sides of the gums along about where the border of the plate comes, are compressed slightly. When the plate is made on the model from this impression and put into the mouth, the margins of the plate are sealed all the way around, and this converts the whole lower surface of the plate into one great suction pad—if we may use that term.

One of the most delicate parts of this operation is the trimming of the plate to just that line that will best seal its borders, for if it is too long it will give the muscles a chance to displace it; if too short, it may allow the air to pass in and thus defeat our whole purpose.—*Dental Practice.*

**A Method of Taking Impressions for Full Dentures by the Use of Modeling Compound and Plaster.**

For full upper impressions, first get a cup that approximately fits the arch. Reaching back over the condyles use soft modeling composition, enough to reach as high as you wish, but not excess enough to disturb the tissues.

Get preliminary impression of mouth and from this preliminary impression make a preliminary model. Mix plaster thick, use a little sulphate of potash, and run model. In a few minutes your model will be hard enough to use.

Now select a cup that will require the least cutting, use a horn mallet, or by bending in any way, shape your cup so as to use the least amount of modeling composition to reach as high as you wish your plate. Leave a little space between the posterior edge of the cup and the roof of the arch.

Now, having the cup properly shaped, place into it some soft modeling composition, and then soapstone your temporary model and take an impression of same, forcing out excess of modeling composition, and trim off excess. Remove model. You now have just enough modeling composition for this impression.

Now comes the most careful part of this work. Place impression in water hot enough to soften composition, place cup in the mouth carefully and square on the arch, hold firmly in position, have patient open the mouth and draw down the lip, so the muscles and frenum will mark the height they will tolerate plate. As soon as composition begins to set, with forefinger reach back to the heel of the cup and press composition against posterior of arch, also around condyles and rim in canine region above cup. After modeling composition sets, remove and chill.

Now mix up some quick-setting plaster, using a little sulphate of potash to the consistency of thick cream. Pour into impression, covering the whole surface, and sling out all but just a coating, and quickly place back into the mouth, and when plaster has set you will have the best fitting impression it is possible for one to get.

Too much plaster in the last effort will spoil the impression.

For lower impression proceed same as above, pressing modeling composition well against the inner side of condyles. —S. D. Potter, *in Summary*.

